# Development and Validation of a Droplet Digital PCR Assay for Detection of Feline Herpesvirus Type-1

**DOI:** 10.3390/vetsci12111107

**Published:** 2025-11-20

**Authors:** Yaxi Zhou, Danni Wu, Mengle Tang, Zihan Ye, Erkai Feng, Haili Zhang, Guoliang Luo, Zhenjun Wang, Chunxia Wang, Lina Liu, Yuening Cheng

**Affiliations:** 1 Key Laboratory of Special Animal Epidemic Disease, Ministry of Agriculture, Institute of Special Animal and Plant Sciences, Chinese Academy of Agriculture Sciences, Changchun 130112, China; 13001757157@163.com (Y.Z.); 13321435868@163.com (D.W.); 17363542534@163.com (M.T.); 18811737750@163.com (Z.Y.); fengerkai@caas.cn (E.F.); osolgl@163.com (G.L.); wangzhenjun@caas.cn (Z.W.); wangchunxia02@caas.cn (C.W.); liulina01@caas.cn (L.L.); 2 State Key Laboratory for Diagnosis and Treatment of Severe Zoonotic Infectious Diseases, Changchun 130062, China; zhanghaili@jlu.edu.cn; 3 Key Laboratory for Zoonosis Research of the Ministry of Education, Institute of Zoonosis, Changchun 130062, China; 4 College of Veterinary Medicine, Jilin University, Changchun 130062, China

**Keywords:** feline herpesvirus type-1, gD gene, droplet digital PCR, real-time quantitative PCR

## Abstract

Feline herpesvirus type-1 (FHV-1), a double-stranded DNA virus, which is a highly infectious upper respiratory tract infection of felids, particularly in kittens. Droplet digital PCR (ddPCR) provides an absolute quantification method with high sensitivity and accuracy. This study aimed to develop a highly sensitive and accurate ddPCR assay for the detection of FHV-1. We designed primers and a probe targeting the FHV-1 glycoprotein D (gD) gene and evaluated the assay’s limit of detection (LOD), sensitivity, repeatability, and specificity in comparison to quantitative real-time PCR (qPCR). The developed ddPCR assay demonstrated a strong linear dynamic range (R^2^ ≥ 0.99) and an exceptionally low LOD of 0.18 copies/μL, which was significantly more sensitive than the method qPCR (LOD ~10 copies/μL). Additionally, the assay exhibited high specificity with no cross-reactivity against other common feline pathogens (feline calicivirus, FCV; feline panleukopenia virus, FPV; feline infectious peritonitis virus, FIPV; *Bordetella bronchiseptica* and *Chlamydia felis*) and displayed outstanding repeatability (inter-run CV < 1.35). When applied to 118 clinical samples, the ddPCR assay achieved a significantly higher positive detection rate (27.4%) compared to qPCR (14.8%). In conclusion, we have successfully established a reliable ddPCR assay for the absolute quantification of FHV-1, providing a superior tool for laboratory diagnosis and research.

## Simple Summary

Feline herpesvirus type-1 (FHV-1) is a highly infectious felid pathogen. This study developed a droplet digital PCR (ddPCR) assay targeting the FHV-1 glycoprotein D (gD) gene for absolute quantification. The ddPCR demonstrated higher sensitivity and specificity compared to quantitative real-time PCR (qPCR), with no cross-reactivity other feline pathogens (FCV, FPV, FIPV, *Bordetella bronchiseptica* and *Chlamydia felis*). Clinical validation showed superior detection performance over qPCR, confirming ddPCR as a reliable tool for FHV-1 diagnosis and research.

## 1. Introduction

Feline upper respiratory tract disease is frequently attributed to co-infections involving feline herpesvirus type-1 (FHV-1) and feline calicivirus (FCV), often accompanied by secondary pathogens such as *Bordetella bronchiseptica* and *Chlamydophila felis* [1]. It is estimated that FHV-1 contributes to approximately half of all diagnosed feline viral upper respiratory tract infections (URTI), although recent surveillance data suggest a declining prevalence of this pathogen [2]. FHV-1 was first isolated in 1958 and widely distributed around the world [3]. Chinese isolates of FHV-1 show high genetic homology with strains from other regions [4]. Cats of all ages are susceptible to FHV-1 infection, with the highest susceptibility observed in kittens between 2 and 4 months of age. Typical clinical manifestations in domestic cats include ocular and nasal discharge, coughing, sneezing, and anorexia. In severe cases, the disease can be life-threatening and may lead to abortion in pregnant cats. As the population of domestic cats continues to grow, the need for rapid and accurate detection of FHV-1 is increasingly urgent.

Currently, the most widely used laboratory diagnostic methods include conventional polymerase chain reaction (PCR) and real-time quantitative PCR (qPCR). Conventional PCR is performed in an open system, making it highly susceptible to contamination, and it only provides qualitative results without quantification. Although qPCR offers higher sensitivity than conventional PCR, it still does not enable absolute quantification and has limited analytical sensitivity for samples with low viral titers. Droplet digital PCR (ddPCR) is a novel absolute quantification technology. Based on the principle of extreme dilution and discrete detection of target nucleic acids, it partitions the reaction system into numerous microscopic droplets or microwells, effectively distributing the PCR mixture into thousands of independent sub-reactions [5,6]. The presence or absence of target template molecules in each partition is detected via fluorescence, and the original concentration of the target nucleic acid is absolutely quantified through Poisson distribution analysis. Compared to qPCR, ddPCR offers higher sensitivity, greater tolerance to inhibitors, and more precise quantification, providing significant advantages for low viral load samples and serving as a valuable complement to qPCR-based detection methods [7,8].

Therefore, the development of a highly sensitive and accurate ddPCR method for FHV-1 is crucial for controlling and preventing the spread of FHV-1 related diseases. However, there is no available ddPCR method for detecting FHV-1 to date. In this study, we developed and validated a highly sensitive ddPCR assay capable of precise quantification of FHV-1.

## 2. Materials and Methods

### 2.1. Virus Strains and Samples Analysis

FHV-1, feline calicivirus (FCV), feline panleukopenia virus (FPV), feline infectious peritonitis virus (FIPV), *Bordetella bronchiseptica* and *Chlamydia felis* were stored in our laboratory at −80 °C. A total of 118 clinical samples (ocular and nasal swabs) from cats exhibiting URTI symptoms were collected across 34 veterinary hospitals located in Jilin province of China, between July 2022 and June 2025.

### 2.2. Primer and TaqMan Probe Design

According to the FHV-1 genomic sequences from GenBank database, the highly conserved sequence of FHV-1 gD gene (Genbank ID: KT963467.1) was aligned using MEGA v11.0.13 software. All primers and a TaqMan probe were designed using Primer Express^®^ v3.0.1, and their specificity was verified through Primer-BLAST (https://www.ncbi.nlm.nih.gov/tools/primer-blast/, accessed on 1 November 2025) screening (Table 1). The same primer–probe set was used for both ddPCR and qPCR assays. All oligonucleotides synthesized by Sangon Biotech (Shanghai, China) Co., Ltd.

### 2.3. Nucleic Acid Extraction

The DNA from FHV-1, FPV, *Bordetella bronchiseptica* and *Chlamydia felis* was extracted with Virus DNA/RNA Fast Kit (TIANGEN, Beijing, China) according to the recommended instructions of manufacturer. The TRIzol reagent (Ambion, Austin, TX, USA) was used to extract viral RNA from the samples of FCV and FIPV following the manufacturer’s guidelines. Reverse transcription was performed using a 1st strand cDNA synthesis kit (Thermo Fisher, Waltham, MA, USA) and total DNA and cDNA concentration were measured and stored at −80 °C until use.

### 2.4. Construction of Standard Plasmids

To generate the standard positive control, the complete gD gene fragment of FHV-1 was amplified by using primers gD-F and gD-R (Table 1) and cloned into the pCE2 TA/Blunt-Zero vector (Vazyme, Nanjing, China). The recombinant plasmid named pCE-FHV-gD was transformed into *E. coli* DH10B and purified by using a Plasmid Miniprep Kit (Omega Bio-tek, Shanghai, China). The concentration of the plasmid was quantified by using the NanoDrop Photometer (Implen GmbH, Gilching, Germany). A ten-fold serial dilution (3.6 × 10^0^–3.6 × 10^4^ copies/μL) of the plasmid in nuclease-free double-distilled water (ddH_2_O) was prepared to plot a ddPCR standard curve. The standard plasmid copy number was calculated using the following formula: Copies/μL = [DNA concentration (ng/μL) × 6.022 × 10^23^]/[Plasmid length (bp) × 660 × 10^9^].

### 2.5. qPCR Assay

The qPCR assay was performed on QuantStudio™ 3 real-time PCR system (Thermo Fisher, Waltham, MA, USA). The reaction volume (20 μL) included: 10 μL of 2 × Taq Pro HS Universal Probe Master Mixa (Vazyme, Nanjing, China), 0.4 μL of each forward and reverse primer (10 μM), 0.2 μL of probe (10 μM), 8 μL of ddH_2_O, and 2 μL of template. The amplifying process was as follows: 95 °C for 30 s, 45 cycles of 95 °C for 10 s and 60 °C for 30 s. After the reaction, a standard curve was plotted, and then the sensitivity, repeatability and specificity tests were conducted. The qPCR assay was performed in triplicate.

### 2.6. Droplet Digital PCR (ddPCR) Assay

The optimal annealing temperature for ddPCR was firstly identified by analyzing temperatures of 56, 58, 60, 62 and 64 °C. The concentration of standard plasmid selected by temperature optimization was 3.6 × 10^4^ copies/μL. Then, the primer-to-probe concentration (500:200, 900:250, 800:300, and 600:400 nM) was optimized. The concentration of standard plasmid was 3.6 × 10^4^ copies/μL. The ddPCR assay was performed in triplicate.

The ddPCR assay was performed in 15 μL volume contained: 3 μL of 5 × ddPCR Mix, 1.35 μL of each forward and reverse primer (10 μM), 0.375 μL of probe (10 μM), 3.925 μL of DNase/RNase-free water, and 5 μL of template. The 14 μL of reaction mixture and 16 μL of oil were loaded onto each of the droplet generation chips to produce droplets on a drop maker. The amplification reaction procedure was as follows: 95 °C for 10 min, 40 cycles of 96 °C for 20 s and 60 °C for 60 s, and ending at 25 °C. Finally, the chips were placed into a droplet reader for fluorescence signal acquisition. The fluorescent signal image from the chip is transmitted to the matching software “GeneADP16” installed on the computer for data processing. According to the Poisson distribution principle, the copy number concentration of the target analyte can be determined simply by counting the number of negative reaction units and the total number of reaction units in the image. Automated Droplet Digital PCR System (AD1617), microfluidic chip kits, and Digital PCR Solution were all procured from Pilot Gene Technology (Hangzhou, China) Co., Ltd.

### 2.7. Analytical Sensitivity and Repeatability of the ddPCR and qPCR

A ten-fold serial dilution of pCE-FHV-gD was obtained using nuclease-free water (3.6 × 10^−1^–3.6 × 10^5^ copies/μL) as the samples to determine the detection limit (LOD). The LOD was defined as the lowest concentration at which ≥95% of technical replicates tested positive and the signal intensity was significantly higher than the background (*p* < 0.01). Parallel qPCR and ddPCR assays were performed synchronously, with each test replicated across three independent sessions to ensure result accuracy. We analyzed standard plasmid dilutions at three concentrations (3.6 × 10^4^, 3.6 × 10^3^ and 3.6 × 10^2^ copies/μL) with the ddPCR method, and inter-assay and intra-assay repeatability tests were performed in triplicate for each respective sample to assess variability in ddPCR.

### 2.8. Specificity of the ddPCR Assay

Specificity tests were performed on FHV-1, FCV, FPV, FIPV, *Bordetella bronchiseptica* and *Chlamydia felis* using the optimized ddPCR assay. The standard plasmid was used at a concentration of 3.6 × 10^4^ copies/μL in nuclease-free water.

### 2.9. Clinical Samples Test

To assess and compare the efficiency of the built ddPCR method for FHV-1 detection with that of qPCR, a total of 118 clinical samples stored in our laboratory were tested. The DNA/cDNA were detected by ddPCR using the optimized protocol described above. The results were compared with those obtained from the qPCR method, which was performed in parallel.

## 3. Results

### 3.1. Development of FHV-1 ddPCR Assay

**Figure 1:**
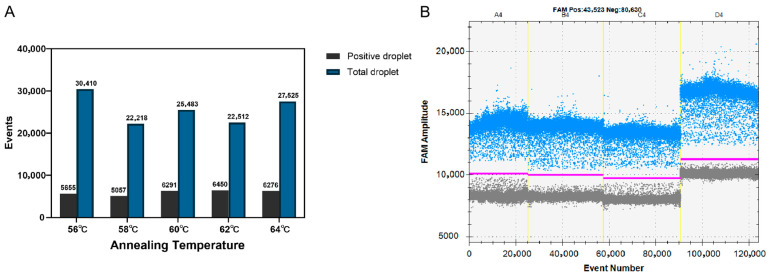
Optimization of FHV-1 ddPCR assay using 3.6 × 10^4^ copies/μL plasmid DNA standard (N = 3 technical replicates per temperature). (**A**) Optimization of annealing temperature. The temperatures evaluated were 56 °C, 58 °C, 60 °C, 62 °C, and 64 °C. (**B**) Optimization of primer and probe concentrations. The tested combinations were as follows: A4: 500 nM primer/200 nM probe; B4: 900 nM primer/250 nM probe; C4: 800 nM primer/300 nM probe; D4: 600 nM primer/400 nM probe. The blue dots are positive droplets, and the grey dots are negative droplets. The red line indicates the fluorescence amplitude threshold for positive/negative calling.

To optimize primer-to-probe concentration and annealing temperature, we evaluated amplification efficiency across temperature gradients (56, 58, 60, 62 and 64 °C) to determine the optimal annealing temperature conditions (Figure 1A). At 62 °C, the fluorescence signal difference between positive and negative droplets was maximized. Therefore, 62 °C was considered the optimal annealing temperature. Next, we tested different primer-to-probe concentrations ratios (500:200, 900:250, 800:300 and 600:400 nM) following the manufacturer’s recommended reaction system and conditions. As shown in Figure 1B, the 900:250 nM ratio (group 2) demonstrated optimal performance by achieving both the highest fluorescence amplitude and clearest separation between positive and negative droplet populations, as quantified by signal-to-noise ratio calculations. The results indicated that the optimal primer and probe concentrations were determined to be 900 and 250 nM, respectively. Therefore, the optimal annealing temperature (62 °C) and primer-to-probe concentration (900:250 nM) were selected as the optimized conditions in further FHV-1 ddPCR assays.

### 3.2. Detection Limits and Repeatability of the ddPCR and qPCR Assays

**Figure 2:**
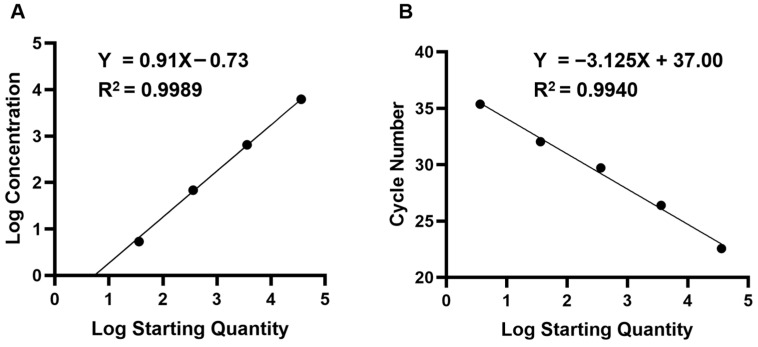
Quantification of serial dilutions of FHV-1 standard plasmid by ddPCR and qPCR. (**A**) Standard curves of FHV-1 plasmids constructed by ddPCR (N = 3). The quantification correlation was obtained by plotting the logarithm of absolute concentration (log_10_ copies/μL) against the log_10_ starting concentration (log_10_ copies/μL), with a regression equation of Y = 0.910X − 0.73 and a coefficient of determination (R^2^) of 0.9989. Error bars represent the standard deviation (SD) of the technical replicates. (**B**) Standard curves of FHV-1 plasmids constructed by qPCR. The standard curve was generated by plotting the quantification cycle (Cq) values against the log starting concentration, with a regression equation of Y = −3.125X + 37.00 and an R^2^ of 0.9940. Statistical analysis of the linearity was performed using linear regression.

We constructed the plasmid pCE-FHV-gD and serially diluted ten-fold for parallel quantification by qPCR and ddPCR established above, statistical analysis demonstrated higher linearity of ddPCR (R^2^ = 0.9989) compared to qPCR (R^2^ = 0.9940) (Figure 2). In Table 2, the LOD of ddPCR was 0.18 copies/μL, which was 55-fold lower than that of qPCR (~10 copies/μL). Furthermore, the results of the last two dilution templates showed no significant change in case of qPCR. The results revealed that the FHV-1 ddPCR method developed in this study exhibits higher sensitivity compared with that of qPCR. In the repeatability tests, the intra-assay coefficient of variation (CV) ranged from 1.20% to 7.35%, and the CV of the inter-assay ranged from 0.44% to 1.35% (Table 3). These results indicated that the developed ddPCR has a good sensitivity, reproducibility and reproducibility.

### 3.3. Specificity of the ddPCR Assay

**Figure 3:**
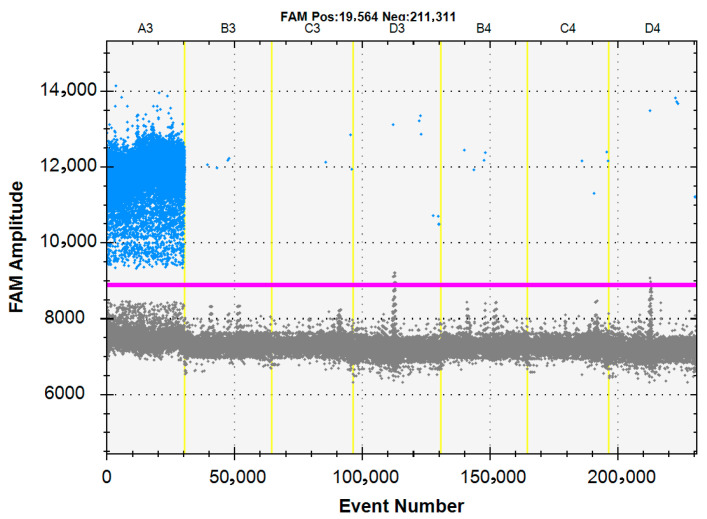
Specificity evaluation of the FHV-1 ddPCR assay. The assay was tested against a panel of samples (N = 3 technical replicates per target) including: A3 FHV-1, B3 FCV, C3 FPV, D3 FIPV, B4 *Bordetella bronchiseptica*, C4 *Chlamydia felis* and D4 no-template control (NTC). The blue dots are positive droplets, and the grey dots are negative droplets. The red line indicates the fluorescence amplitude threshold for positive/negative calling. All non-target pathogens and NTC showed no cross-reactivity, demonstrating high assay specificity.

To validate the specificity of the ddPCR assay, DNA/cDNA templates from FHV-1, FCV, FPV, FIPV, *Bordetella bronchiseptica*, *Chlamydia felis* and nuclease-free water were tested. The results demonstrated that only FHV-1 generated positive fluorescence signals, while other pathogen tests were negative, confirming the high specificity of the established ddPCR method (Figure 3).

### 3.4. Clinical Samples Tests

Clinical sample analysis was performed on 118 feline specimens presenting URTI symptoms, collected across 34 veterinary clinics in Jilin Province, using the optimized FHV-1 ddPCR and qPCR assays developed in this study. As shown in Table 4, FHV-1 ddPCR exhibited a 27.4% positive detection rate (23 of 118), while the qPCR detection rate was only 14.8% (12 of 118). All FHV-1 positive amplicons were further sequenced by Sangon Biotech (Shanghai, China) Co., Ltd., confirming exclusive amplification of the target gD region. According to these test data, quantitative analysis revealed significantly higher detection rates in positive samples by ddPCR compared to qPCR. Based on the binomial distribution, the exact *p*-value for the discordant pairs (B = 0, C = 11) was calculated to be 0.001, indicating a statistically significant difference in the positive detection rates between the two assays. The observed agreement (P_o_) was (12 + 95)/118 ≈ 0.9068, while the expected agreement (P_e_) was approximately 0.7398. The Cohen’s Kappa value was approximately 0.64, with a 95% confidence interval of (0.523, 0.761), suggesting moderate to good agreement between the two methods. The Positive Percent Agreement (PPA) was 12/(12 + 0) × 100% = 100%, and the Negative Percent Agreement (NPA) was 95/(95 + 11) × 100% ≈ 89.62%.

## 4. Discussion

The concept of digital PCR (dPCR) originated in the 1990s as a third-generation technology evolving from qPCR. However, it was not widely adopted until the first commercial instrument was introduced in 2007. Droplet digital PCR technology achieved commercialization in 2011 and has since been extensively applied in clinical settings for the detection and quantification of various pathogens [9,10,11,12]. Currently, dPCR is primarily applied for rare mutant genes detection [13,14], viral load quantification [15,16,17], and precise copy number quantification (CNV and NGS libraries) [18], etc. It is also recognized as a promising monitoring tool for diseases such as cancer and serves as a suitable method for microbial source tracking [19,20].

dPCR technology involves partitioning nucleic acids into a large number of independent reaction units for individual amplification. By statistically analyzing the number of reaction units containing the target sequence (considered positive) and those without the target sequence (considered negative), absolute quantitative detection of the target molecule is achieved. During actual data analysis, reaction units containing more than one template may occur. In such cases, the Poisson distribution is applied to correct and compensate the count of positive units, obtaining the average nucleic acid copy number per reaction unit, enabling precise quantification. Commercially available dPCR systems can be categorized into two types based on their sample partitioning and detection methods. The first category is chamber-based digital PCR (cdPCR) systems, which utilize physical structures as partitioning units. The second category is droplet digital PCR systems that perform PCR in water-in-oil droplet emulsions, capable of generating up to 10 million partitions. Step-emulsification systems address this through massive parallelization, enabling the production of tens of thousands of droplets within minutes while maintaining relatively uniform droplet size [21]. Compared to qPCR, the ddPCR demonstrates enhanced sensitivity and accuracy in detecting low copy numbers and subtle template variations, while enabling absolute quantification without standard curves [4]. However, when processing samples with high concentrations, droplet saturation (overload) may occur beyond the detection limit, resulting in false-negative fluorescence signals and revealing the inherent upper detection limit of ddPCR. Our experiments showed that over-saturated wells are automatically flagged as “NaN-NaN” by the ddPCR analysis software, indicating that fluorescence signals exceed the linear detection range and cannot be used for concentration calculation. This software labeling mechanism provides a direct visual cue for identifying problematic samples, but it also highlights a practical challenge: for diagnostic samples with unknown input concentrations, the need to retest after dilution may increase operational complexity and turnaround time. We acknowledge that simply recommending “appropriate dilution” for samples approaching saturation, as previously noted, is insufficient to fully mitigate the risk of false negatives in strongly positive samples. To address this, we propose a combined strategy of software monitoring and gradient dilution. When over-saturated wells are detected, samples are subjected to 2–3 parallel dilutions (e.g., 1:10, 1:100, 1:1000) to verify consistency of positive signals across dilution gradients, thereby reducing false negatives caused by signal overload. As ddPCR quantification is fundamentally based on Poisson distribution principles, concentrations exceeding this threshold cannot be statistically corrected, thereby compromising quantitative accuracy. Consequently, when validating high-concentration samples, they require diluting before test. The high-concentration samples still tested positive, which did not influence the qualitative diagnosis.

The results demonstrate that ddPCR exhibits significantly higher sensitivity than qPCR, with a LOD of 0.18 copies/μL. The intra-assay and inter-assay CV ranged from 1.20% to 7.35% and 1.10% to 1.35%, indicating high robustness and repeatability of the ddPCR system. Furthermore, ddPCR detection showed high specificity, without positive signals observed against other common feline pathogens (such as FCV, FPV, FIPV, *Bordetella bronchiseptica* and *Chlamydia felis*). Although qPCR is commonly used for FHV-1 detection, ddPCR effectively identifies low viral loads, making it more suitable for early detection of FHV-1 infection. Notably, the established FHV-1 ddPCR assay demonstrated the presence of several positive droplets in negative samples, indicating that the limit of blank could not achieve 0 copies/μL. Furthermore, sensitivity evaluations revealed suboptimal precision at lower copy number concentrations. These observations suggest the need for further optimization of the assay protocol.

## 5. Conclusions

In conclusion, this study developed and validated a ddPCR assay for the absolute quantification of FHV-1 within the defined dynamic range. Compared with qPCR, the ddPCR assay demonstrated superior sensitivity for detecting low viral loads and showed high reproducibility, showing potential as a valuable tool for early detection of FHV-1 infection. It is important to note that the assay’s performance is characterized by a non-zero limit of blank, reduced precision at the lower limit of quantification, and a specificity panel that warrants further expansion. In clinical applications, ddPCR detected additional positive cases missed by qPCR, suggesting its utility as a complementary method for FHV-1 monitoring and epidemiological studies.

## Figures and Tables

**Table 1:** Primer and probe sequences.

Primer/Probe	Sequence (5′→3′)	Amplicon Size
gD-F	TGAGTGGTGGTGTGTGGCATAT	1125 bp
gD-R	ACCGCCCACTTCATATCTTCCC	
dd/qPCR-gD-F	GAACCCCCAGTAAGATTCCA	80 bp
dd/qPCR-gD-R	GACCGTTTCGAATCCTCACT	
dd/qPCR-gD-Probe	FAM-CCATTCGATATGATCGTCCCGCC-BHQ1	

**Table 2:** Detection limits of qPCR and ddPCR using diluted FHV-1 plasmid.

Concentration of Standard Plasmid (Copies/μL)	qPCR (Mean Cq * Value)	ddPCR (Mean Copies/μL ± SD ^#^)
3.6 × 10^5^	21.08	Overload ^##^
3.6 × 10^4^	24.25	6128.63 ± 69.66
3.6 × 10^3^	27.49	644.46 ± 9.23
3.6 × 10^2^	30.40	68.56 ± 1.35
3.6 × 10^1^	32.84	5.37 ± 0.18
3.6 × 10^0^	33.12	0.75 ± 0.03
3.6 × 10^−1^	33.80	0.18 ± 0.01
NTC **	ND ***	ND ***

* Cq: quantification cycle; ** NTC: no-template control; *** ND: not detected; ^#^ SD: standard deviation; ^##^ Overload: “Overload” indicates a quantitative anomaly in ddPCR caused by excessively high target DNA concentration. When the concentration of the target sequence in the sample exceeds the upper limit of the detection system, numerous droplets will contain two or more target molecules, thereby violating the quantitative principles of ddPCR based on “single molecule distribution” and Poisson distribution, making it impossible for the software to calculate the concentration of the target accurately.

**Table 3:** Robustness and reproducibility analysis of FHV-1 ddPCR.

Concentration of Standard Plasmid (Copies/μL)	Intra-Assay Variation (Robustness)			Inter-Assay Variation (Reproducibility)		
	Mean * (Copies/μL)	SD ^#^	CV ^†^ (%)	Mean * (Copies/μL)	SD ^#^	CV ^†^ (%)
3.6 × 10^4^	6128.63	74.93	1.20	6175.40	68.01	1.10
3.6 × 10^3^	644.46	16.27	2.52	642.93	2.83	0.44
3.6 × 10^2^	68.56	5.04	7.35	68.93	0.93	1.35

* Mean: the average copy number per reaction (copies/μL); ^#^ SD: standard deviation; ^†^ CV: coefficient of variation, calculated as (SD/Mean) × 100%. All measurements were performed in accordance with the optimized ddPCR protocol described in Methods. Data are derived from N = 3 independent ddPCR reactions for intra-assay variation (robustness) and N = 3 separate experimental runs for inter-assay variation (reproducibility) at each plasmid concentration.

**Table 4:** Comparison of ddPCR and qPCR sensitivity for FHV-1 clinical samples.

qPCR	ddPCR		Total
	Positive	Negative	
Positive	12	0	12
Negative	11	95	106
Total	23	95	118

## Data Availability

The original contributions presented in this study are included in the article. Further inquiries can be directed to the corresponding author.
